# Application of a sepsis flow chip (SFC) assay for the molecular diagnosis of paediatric sepsis

**DOI:** 10.1099/acmi.0.000474.v4

**Published:** 2023-04-21

**Authors:** Dimas Seto Prasetyo, Mulya Rahma Karyanti, Irene Yuniar, Yulia Rosa Saharman, Livya Holiwono

**Affiliations:** ^1^​ Clinical Microbiology Medical Staff, Cipto Mangunkusumo General Hospital, Jakarta, Indonesia; ^2^​ Department of Microbiology Faculty of Medicine, Universitas Indonesia, Jakarta, Indonesia; ^3^​ Pediatric Child Health Medical Staff, Cipto Mangunkusumo General Hospital, Jakarta, Indonesia; ^4^​ Department of Child Health Faculty of Medicine, Universitas Indonesia, Jakarta, Indonesia

**Keywords:** amplification-hybridization, paediatric sepsis, blood culture, antimicrobial stewardship

## Abstract

A delay in detecting sepsis pathogens is a problematic issue for determining definitive antibiotic therapy for the causative pathogens. The gold standard method for sepsis is blood culture but this requires 3 days to detect the definitive pathogen. Molecular methods offer rapid identification of pathogens. We evaluated the use of sepsis flow chip (SFC) assay for identifying pathogens from children with sepsis. Blood samples from children with sepsis were collected and incubated in a culture device. Positive samples were subjected to amplification-hybridization using SFC assay and culture. A total of 94 samples from 47 patients were recovered, from which 25 isolates were recovered, including *Klebsiella pneumoniae* (11) and *Staphylococcus epidermidis* (6). From 25 positive blood culture bottles subjected to SFC assay, 24 genus/species and 18 resistance genes were detected. The sensitivity, specificity and conformity was 80, 94.2 and 94.68 % respectively. SFC assay offers promise to identify pathogens from positive blood culture in paediatric patients with sepsis and may support the antimicrobial stewardship programme in hospitals.

## Data Summary

No data were generated or reused in the research. The authors confirm that all supporting data, code and protocols have been provided within the article.

**Impact Statement**

This paper discusses the application of a molecular method in diagnosing paediatric patients with sepsis. The use of this molecular method in diagnosing paediatric sepsis may help physicians to give appropriate antibiotics since it can give rapid results in identifying pathogens while waiting for definitive antibiotic susceptibility testing. Thus, it could help the antimicrobial stewardship programme in hospitals.

This paper may represent a way to start a similar multi-centre study in Indonesia for nationwide genomic antimicrobial resistance surveillance.

## Introduction

Sepsis is becoming a problematic issue [1]. In 2013, the World Health Organization reported that there were approximately 6.3 million deaths related to sepsis in children <5 years old, with the majority of cases found in developing countries [2]. In 2019, the prevalence of septic shock among children admitted to a referral hospital in Kenya was 15.4 % [3]. Another report from Myanmar showed that from 2017 to 2019, the prevalence of suspected neonatal sepsis was 15.6 % [4]. A 2016 report from Sardjito Hospital, Yogyakarta, Indonesia, showed that the mortality rate among children with sepsis in an intensive care unit (ICU) was 88.2 % [5]. In Dr. Cipto Mangunkusumo Hospital, Jakarta, Indonesia, the prevalence of sepsis among children in a paediatric ICU in 2019 was 20.8 % [6].

A main problem regarding paediatric sepsis is that in almost half of cases, the definitive pathogens cannot be identified [7]. In addition, there is no one ideal laboratory test that can detect sepsis accurately [8]. Blood culture as the gold standard does not give satisfactory results, as the final identification and antibiotic susceptibility testing results are only available 3–4 days after sample collection [9]. A delay in defining the definitive pathogen and antibiotic therapy means the patient may be exposed to a wide spectrum of antibiotics that can lead to an increase in the resistance rate [10].

Advanced molecular methods offer rapid bacterial identification and resistance gene detection [11]. The rapid results from these methods may help clinicians in terms of changing the antibiotic therapy according to the species and resistance genes detected [12]. There are two types of molecular assays that can be used in diagnosing sepsis: first, assays that can directly use whole blood for the specimen, and second, assays that use positive blood cultures. The latter methods offer relatively higher sensitivity and specificity than the former because the level of bacteria may have been increased after incubation in the blood culture machine [13].

Several papers have reviewed the use of molecular techniques in aiding the diagnosis of sepsis. Pammi *et al*. reported that the overall sensitivity and specificity of molecular methods in establishing neonatal sepsis were 90 and 96 %, respectively [14]. Sinha *et al*. reviewed several emerging methods in diagnosing sepsis with sensitivity and specificity in the range 11–87  and 69–96 %, respectively [10]. Oeser *et al*. found that real-time PCR assays may have benefit in the diagnosis of neonatal sepsis [15]. Zacharioudakis *et al.* reported that the use of molecular diagnostic assays may be cost-effective in stopping patients from receiving inappropriate antibiotic therapy [16]. Nevertheless, the utility of molecular assays in paediatric sepsis in Indonesia has not been explored widely.

We performed an amplification–hybridization molecular method from children diagnosed with sepsis. This method, namely the Hybrispot sepsis flow chip (SFC; Master Diagnostica), is a microarray-based assay which can detect up to 21 genus/species of various species of bacteria and fungi from positive blood cultures. Beside species identification, it can also detect up to 20 genes conferring resistance to several antibiotic classes, including cephalosporins and carbapenems [17]. The objective was to evaluate the performance of the SFC in establishing a diagnosis of sepsis in a paediatric setting.

## Methods

### Study design, ethical approval and patient selection

This was a prospective cross-sectional diagnostic study comparing the SFC assay with conventional blood culture identification and antibiotic susceptibility testing. The inclusion criteria were children aged 0–18 years old, diagnosed with suspected sepsis, from an infection ward and ICU at Dr. Cipto Mangunkusumo Hospital, Jakarta. After receiving informed consent from the parents, we collected a pair of blood culture samples. The blood culture bottle was then delivered to the Clinical Microbiology Laboratory, Faculty of Medicine, Universitas Indonesia. Ethical approval was obtained from The Ethics Committee of the Faculty of Medicine, Universitas Indonesia, number KET-898/UN2.F1/ETIK/PPM.00.02/2019. Research permission was granted from the Research Unit of Dr. Cipto Mangunkusumo Hospital. The number of samples necessary to give a significant result was estimated at 26.

### Specimen collection and conventional culture

A pair of blood culture bottle samples were drawn from patients with sepsis from the emergency, paediatric intensive care unit (PICU) and infection ward from January to December 2020. The blood culture bottles were incubated in a BacT/Alert blood culture machine (bioMérieux) until they produced a positive signal. Positive blood cultures were subjected to Gram staining and media inoculation. Colonies were identified and tested for antibiotic susceptibility using the Vitek-2 Compact system (bioMérieux). For negative blood cultures, after 5× incubation for 24 h, the sample was also subjected to Gram staining and inoculated in blood agar to confirm the negative result.

### SFC assay

The SFC assay was performed using an aliquot of 500 µl from each positive and negative blood culture bottle. This aliquot of 500 µl was then diluted 1 : 10 using normal saline and as much as 4 µl of the 10^−1^ dilution was used as a template for the SFC assay, according to the manufacturer's instructions [18]. In brief, the SFC assay consisted of two steps: first, the template underwent a 40-cycle PCR for approximately 2 h; and second, the PCR product was then hybridized on a chip which contained several probes useful for species identification and resistance gene detection. The hybridization result was visualized using the instrument's camera. This SFC assay can detect up to 21 genus/species of bacteria and fungi, such as *Stenotrophomonas maltophilia* and *Candida albican*s. Resistance gene detection included the most common antibiotic resistance genes, such as *mecA*, β-lactamase genes [carbapenemases and extended-spectrum beta-lactamases (ESBLs)] and vancomycin-restance genes (*vanA* and *vanB*).

### Data and statistical analysis

The sensitivity, specificity, positive predictive value, negative predictive value and conformity of the SFC were measured using a 2×2 table as the final outcome. Statistical analysis was conducted using a chi-square and Mann–Whitney test.

## Results

From January to December 2020, a total of 94 blood culture samples were collected from 47 patients. The patient’s age ranged from 0 to 17 years. Of 94 samples, 25 isolates detected by the Vitek-2 Compact (blood sample positivity was 26.59 %) were identified, as shown in Table 1.

**Table 1.** Organisms identified using the Vitek-2 Compact

No	Organism	N (%)
1	* Klebsiella pneumoniae *	11 (44)
2	* Staphylococcus epidermidis *	6 (24)
3	*Candida parapsilosis*	3 (12)
4	*Enterobacter cloaceae*	2 (8)
5	* Staphylococcus saprophyticus *	2 (8)
6	*Candida albicans*	1 (4)
	Total	25 (100)

From 25 positive blood culture samples, the Hybrispot SFC yielded 24 organisms and 18 resistance genes, as shown in Tables 2 and 3, respectively. For each gene, the prevalence of ESBL *CTX-M*, carbapenemase *NDM*, ESBL *SHV*, methicillin-resistant (*mecA*) and carbapenemase *GES* were 28.89, 26.67, 17.78, 11.11 and 2.22 %, respectively. The comparison between the Hybrispot-12 SFC and Vitek-2 Compact identification result is shown in Table 4. Based on Table 4, the sensitivity, specificity and positive likelihood ratio for the Hybrispot-12 SFC were 80 % [95 % confidence interval (CI) 58.7–92.4 %], 94.2 % (95 % CI 85.1–98.1 %) and 83.3 % (95 % CI 61.8–94.5 %), respectively. The positive and negative predictive value was 83.33 and 92.85 %, respectively. The overall conformity for the Hybrispot-12 SFC was 90.43 %.

**Table 2.** Organisms identified using the Hybrispot-12 SFC

Organism	N (%)
* Enterobacteriaceae *, *Klebsiella pneumoniae *	11 (45.83)
* Staphylococcus* spp	5 (20.83)
* Pseudomonas aeruginosa *	2 (8.3)
*Candida* spp	2 (8.3)
* Enterobacteriaceae* (other than *K.pneumoniae*)	2 (8.3)
* Proteus* spp	1 (4.2)
* Streptococcus pneumoniae *	1 (4.2)
Total	24 (100)

**Table 3.** Resistance genes detected by the Hybrispot-12 SFC

Organism	Resistance gene	N (%)
*K.pneumoniae*	ESBL SHV, ESBL CTX-M, Carbapenemase NDM	7 (29,2)
* Staphylococcus* spp.	Methicillin Resistant (mecA)	4 (16,6)
* Proteus* sp	Methicillin Resistant (mecA)	1 (4,2)
* E. cloacae *	ESBL CTX-M, Carbapenemase NDM	2 (8,3)
* K. pneumoniae *	ESBL CTX-M, Carbapenemase NDM	2 (8,3)
* K. pneumoniae *	ESBL CTX-M	1 (4,2)
* K. pneumoniae *	ESBL SHV, ESBL CTX-M, Carbapenemase GES, Carbapenemase NDM	1 (4,2)
* P. aeruginosa *, *Candida* spp, *S. pneumoniae *, *Staphylococcus* spp	Not detected	6 (25)
	Total	24 (100)

**Table 4.** Comparison between Hybrispot-12 SFC and Vitek-2 Compact

Hybrispot-12 SCF	Vitek-2 Compact	
	Positive	Negative
Positive	20	4
Negative	5	65
Total	25	69

Based on Tables 1 and 2, there was a difference in species identification between the conventional (Vitek-2 Compact) and SFC assay because in the SFC, there is, for example, no probe for yeast identification other than *Candida albicans*. Also, no specific probe exists for identification of *Staphylococcis epidermidis* and *Staphylococcis saprophyticus*, and hence the isolates were identified as *Staphylococcus* sp. in the SFC. Some taxa were detected by the SFC but not detected in conventional culture, such as *Pseudomonas aeruginosa *, *Proteus* spp. and *Streptococcus pneumoniae *. Isolates that were identified in conventional culture but not detected in SFC included *Candida parapsilosis*, *Candida albicans* and *Staphylococcus epidermidis *.

Concerning the time between sample collection and results, for all blood cultures (94 samples), the median time for the Hybrispot-12 SFC and Vitek-2 Compact was 5 and 6 days, respectively. For positive blood culture samples (25 samples), the median time between sample collection and result release for the Hybrispot-12 SFC and Vitek-2 Compact was 1 and 4 days, respectively (*P*<0.05).

## Discussion

To our knowledge, this is the first study comparing an amplification–hybridization assay, namely Hybrispot-12 SFC, to conventional blood culture in diagnosing paediatric sepsis in Indonesia. A previous study reported the use of the Hybrispot-12 SFC in identifying microorganisms from positive blood cultures in adult patients [17].

Sepsis, as a major health problem, especially in the paediatric field, requires rapid identification of the pathogens to administer appropriate antibiotics [19]. Blood culture as the gold standard method for diagnosing sepsis has limited sensitivity. Culture results are generally available 3–5 days after sample collection, so that patients are at risk of being exposed to long-term empirical antibiotic therapy [9]. This could be mitigated by implementing a rapid molecular diagnostic assay for sepsis patients [20].

In this study, we observed that the positivity of blood cultures was around 26 %. This was higher than from a report in Germany (18.6 %) [21]. However, our finding was still lower than from another study in Nepal which reported that blood culture positivity among newborns with sepsis was 32.4 % [22]. Meanwhile, a study in Pakistan reported that blood culture positivity among paediatric septicaemia patients was 36 % [23]. We did not measure positivity for the SFC as it was only validated for positive blood culture bottle samples.

Our study revealed that *K. pneumoniae* was the most common pathogen isolated from a positive blood culture from a patient with sepsis (44 %). This was higher than from a report from Dr. Soetomo Hospital, Surabaya, which revealed that 18.4 % of all organisms isolated from positive blood culture specimens were *K. pneumoniae* [24]. Our finding could be higher because we collected not only from the PICU but also from other wards, such as emergency and neonatal intensive care unit (NICU) wards. A different finding was reported in Nepal, which showed that more than 50 % of organisms isolated from blood from patients in an NICU were *S. aureus* [22].

Similar assays, such as BioFire Film Array (bioMérieux) and Luminex Verigene (Luminex), have been developed to identify microbes at the genus or species level and also able to detect several antimicrobial resistance markers [25, 26]. The Luminex Verigene includes two separate panels for each Gram-positive and Gram-negative bacterium [27] while the SFC only includes one chip panel that covers both important Gram-positive and Gram-negative bacteria [18]. The number of genera and species that can be detected by the SCF is similar to the BioFire FilmArray Blood Cultur Identification (BCID) but the latter can identify *Candida glabrata*, *Candida parapsilosis*, *Candida krusei* and *Candida tropicalis* [26] whereas the SFC can only identify *Candida albicans* and *Candida* sp. [18]. In terms of resistance markers, all of the above-mentioned systems are able to detect similar resistance genes, such as *blaKPC*, *blaNDM*, *blaVIM*, *blaCTX-M*, *mecA*, *vanA* and *vanB* [18, 27, 28].

Our study found that the SFC showed promising sensitivity and specificity. The result was comparable to conventional culture methods. The main advantage of the SFC was the time needed to obtain a species identification and resistance gene detection. A result could be obtained approximately 4–6 h after the blood culture showed a positive signal [18]. A study from Sao Paolo, Brazil, showed that the use of a molecular assay gave a significantly shorter time for antibiotic adjustment than conventional blood culture and shorter duration of antibiotic therapy [29]. The molecular assay may help with regard to the diagnostic inefficiency in neonatal sepsis, which requires a rapid assay with high sensitivity and specificity [30]. A possible challenge in applying the molecular assay was that it needed a sophisticated laboratory, and costs may be quite high [31].

A limitation of this study was that we did not conduct a cost-effective analysis since we did not compare test costs between the Hybrispot-12 SFC and conventional culture. In addition, we did not measure the clinical outcome and impact of the Hybrispot-12 SFC result in changing from empiric to definitive antibiotic therapy in our patients. Another limitation was that we did not compare between this chip and another similar molecular-based assay. Further study should be done to compare all point-of-care rapid molecular tests in the paediatric sepsis setting. Nevertheless, use of the Hybrispot-12 SFC may support the antimicrobial stewardship programme by providing rapid identification and resistance gene detection, given that we found that among 12 isolates containing the carbapenemase *NDM* gene, 10 isolates were phenotypically resistant to carbapenem. Further research should be done to analyse the cost-effectiveness of the SFC assay compared to conventional culture.
